# Report of Five Cases of Typhus Fever Treated with Belladonna

**Published:** 1871-07

**Authors:** T. S. Latimer


### REPORT OF FIVE CASES OF TYPHUS FEVER TREATED WITH BELLADONNA.

BY DR. T. S. LATIMER.

A few months ago my attention was arrested by an article in the "London Medical Times and Gazette" on the use of belladona in typhus fever. So signal was the benefit ascribed to it by the writer, that I determined to try it at the first opportunity, for which I had not long to wait.

On the 29th of April I was called to see a negro girl, about 25 years old, living in an alley immediately in the rear of the jail, where, at that time, there were quite a number of cases of typhus. Though I could trace no direct communication with any one who had had the disease, the proximity of the jail and the filthiness of he neighborhood sufficiently accounted for its origin. I have had onsiderable familiarity with this disease in hospital practice, and had no difficulty in diagnosing the case before me. I shall not attempt to describe the symptoms at length; they were such as ordinarily characterize the disease, presenting no especial peculiarity. A few days of languor and lassitude, with dull headache, associated with constipation, preceded a slight chill, with subsequent fever, increase of headache, intellectual dullness, with a restless sense of danger giving to the face that peculiarly anxious expression quite characteristic of the disease, which gradually gave place to an expression of weary indifference, were the prominent symptoms in each of these cases. This girl had been confined to her bed two days when I saw her. She was in a filthy condition, as was everything else in the room. I ordered all her clothing to be changed, her whole body to be well washed, a mattress to be placed on the floor, on which she was put, and all other articles of bedding and wearing apparel to be removed from the room; that the whole house should be thrown open as freely as the weather would permit, and that every person living in the house should take a morning and evening bath. I then directed $\frac{1}{4}$ gr. of the extract of belladonna every four hours, the free use of beef tea, and that the whole surface of the body should be sponged with tepid water twice a day. This treatment was continued without modification of any kind until the 5th day of May, when I directed, in addition, one-half ounce of brandy every four hours. The patient did not become delirious until the seventh day, and the delirium began to subside about the tenth day; at no time was it very great. It was possible at all times to fix her attention and obtain intelligent answers to plain questions. By the twelfth day I believed her out of danger, and declared her convalescent. On the tenth day of her illness, the eighth of its administration, the belladonna was directed to be given but twice a day, and on the twelfth day it was stopped altogether, as was also the alcoholic stimulant, except that the yolk of an egg was occasionally given, beaten up with a tablespoonful of brandy, which proved a palatable, nutritious, and readily-digested article of diet. A rapid convalescence ensued, without the occurrence of retarding circumstances.

On the 12th of May a little daughter of the first patient, about six years of age, became affected in the same manner, and was put on the same treatment, the dose of belladonna being, of course, much diminished, (1-40 gr. every four hours.) The strength began to fail a little earlier than in the former case, and it was necessary to use the alcoholic stimulant more freely. In no other respect did this case differ markedly from the first. Convalescence began about the same period, and progressed favorably to recovery.

On the 18th of May a negro woman, who had nursed the first case, was attacked, and the disease pursued the same course, with little or no variation, except that there was a little more prostration, and convalesence did not begin quite so soon, nor progress so rapidly as in the other cases, owing, I have no doubt, to the fact that she was a delicate phthisical woman. The treatment was the same, except that in addition to the stimulant, which was used in the same quantity and with the same frequency, the yolk of an egg beaten up with brandy was taken twice a day. She seemed to enjoy the egg, and no unpleasant effect was observed from it.

On the same day (May 18th) I was called to see a white woman, æt. 38, who had had a slight chill on the previous day, was now suffering with violent headache and pain in the back, and indeed with very much the same train of symptoms as the cases just related; but I did not suspect the true nature of the disease, owing to the fact that she had had a large abscess in the submaxiliary region, which had opened into the mouth a few days before. She having accidentally swallowed a part of the pus was intensely nauseated, and vomited freely. I was therefore disposed to refer the condition of this patient to pyemia, thinking that a portion of the pus had been absorbed from the stomach, or perhaps from the seat of the abscess. Nor did I suspect the true nature of the disease until the seventh day, when her mother called my attention to an abundant eruption upon her body, which I at once recognized as the characteristic eruption of typhus. Up to this time I had been treating her with quinine and iron, with alcoholic stimulants, to which I now made the addition of the extract of belladonna, given with the same frequency and in the same quantity as in the other cases. The egg and brandy, and beef tea were freely given. This patient was confined to her bed about three weeks, and her convalesence has never been perfect. She is a phthisiual subject, and that disease has made very rapid progress since the inception of typhus, and I think she has but a few months to live.

On the 2nd of June a negro woman, who had been living in the same house with the first three patients during their entire illness, but had subsequently removed to another neighborhood, became affected with the same disease, which pursued a course differing so little from those already related that no especial description is necessary. The belladonna was again administered, and the brandy for three or four days, during the most critical period of the disease, was given in tablespoonful doses, four times a day. She is now (June 27th) quite well, though of course she has not yet recovered her ordinary strength, and still has some stiffness and soreness about the joints.

In none of these cases could I observe any definite or special effect from the belladonna; but I am strongly inclined to believe it exerted a favorable influence on the general course of the disease, and somewhat lessened the severity and duration of the delirium. In neither case was any other medicine whatever used, except in that mistaken for pyemia, in which the quinine and iron were continued throughout the disease. The quantity of stimulus used was small, and that but for a short time, except where the phthisical complication existed. I am, however, disposed to refer considerable benefit to the frequent sponging, which certainly added greatly to the comfort of the patients and, I believe, to their recovery.—*Med. and Surg. Reporter.*

